# Integration of 24 Feature Types to Accurately Detect and Predict Seizures Using Scalp EEG Signals

**DOI:** 10.3390/s18051372

**Published:** 2018-04-28

**Authors:** Yinda Zhang, Shuhan Yang, Yang Liu, Yexian Zhang, Bingfeng Han, Fengfeng Zhou

**Affiliations:** College of Computer Science and Technology, and Key Laboratory of Symbolic Computation and Knowledge Engineering of Ministry of Education, Jilin University, Changchun 130012, China; zhangyd2115@mails.jlu.edu.cn (Y.Z.); shyang17@mails.jlu.edu.cn (S.Y.); ly16@mails.jlu.edu.cn (Y.L.); zhangyx17@mails.jlu.edu.cn (Y.Z.); hanbf2115@mails.jlu.edu.cn (B.H.)

**Keywords:** EEG, epilepsy, seizure detection, seizure prediction, feature engineering, feature selection, SVM, classification

## Abstract

The neurological disorder epilepsy causes substantial problems to the patients with uncontrolled seizures or even sudden deaths. Accurate detection and prediction of epileptic seizures will significantly improve the life quality of epileptic patients. Various feature extraction algorithms were proposed to describe the EEG signals in frequency or time domains. Both invasive intracranial and non-invasive scalp EEG signals have been screened for the epileptic seizure patterns. This study extracted a comprehensive list of 24 feature types from the scalp EEG signals and found 170 out of the 2794 features for an accurate classification of epileptic seizures. An accuracy (Acc) of 99.40% was optimized for detecting epileptic seizures from the scalp EEG signals. A balanced accuracy (bAcc) was calculated as the average of sensitivity and specificity and our seizure detection model achieved 99.61% in bAcc. The same experimental procedure was applied to predict epileptic seizures in advance, and the model achieved Acc = 99.17% for predicting epileptic seizures 10 s before happening.

## 1. Introduction

Epilepsy is one of the most common neurological disorders and affects 60 million people around the world [1,2]. Active epilepsy patients are 4 to 8 among 1000 in the population [3], and may reach as high as 57 among 1000 in some developing countries [4]. Unattended epilepsy patients may suffer sudden unexpected death due to various factors, including epilepsy associated syndromes [5] and uncontrolled generalized tonic–clonic seizures [6], etc. Surgery may help cure the epilepsy symptoms [7] but the success rate needs to be increased [8]. Detection or prediction of seizure occurrences may be established using Electroencephalography (EEG) signals [9] or other non-invasive biomedical measurements [10], and will provide a better monitoring for the unattended epilepsy patients.

EEG is a technology used to record the electrical signals produced by the brain neurons and the clinical EEG recorder usually takes 128 channels of electric probes [11]. The voltage fluctuations in an EEG channel are associated with the neural activities and are hypothesized to have close relationships with various brain activities [12]. This hypothesis serves as the basis of many EEG-based applications, e.g., detection of epileptic seizures [13] or sleep stages [14]. EEG has already become one of the major technologies to monitor and detect the epilepsy occurrences due to its nature of non-invasiveness and low-cost [15].

Epileptic seizures may be detected by experienced clinicians’ manual screening of EEG signals but its very labor intensive and time consuming [16]. A modern EEG recorder may generate as many as 1000 data points per second (1000 Hz in sampling rate), and a standard recording procedure may last for a few days [16]. One day of such a recording procedure will generate 11 billion data points, which renders the manual screening highly labor intensive. Many clinical studies demonstrated that epilepsy specific waveforms, like spikes and sharp waves, appear during the epileptic seizure onset, or even in a short time before the onset [17]. This forms the basis of in silico detection and prediction algorithms of epileptic seizures.

Various algorithms were proposed to extract features from the EEG signals for training the epileptic seizure diagnosis models. An EEG-based epileptic seizure diagnosis model evaluates whether an EEG signal is more similar to that of a seizure onset event than an inter-ictal EEG signal. This EEG signal will be predicted as an epileptic seizure onset or inter-itcal status, based on how it is similar to the EEG signals of seizure or inter-ictal statuses. It’s intuitive to extract characteristic features directly from the time domain data, e.g., the inter-channel Robust Generalized Synchrony features [18]. Another widely-employed strategy is to extract the frequency domain features from the given EEG signals. Fourier Transform is one of the most common algorithms to extract the frequency domain features from time-series data and researchers usually use the approximate implementation Fast Fourier Transform (FFT) [19,20]. But FFT or similar algorithm work on stationary signals and do not provide time information for the extracted features. Short Time Fourier Transform (STFT) was proposed to solve this issue by calculating the fourier transformed spectrum within a series of sliding windows, so that both time and frequency domain information were reflected in the STFT features [21,22]. The integration of both time and frequency domain features was shown to outperform the epileptic seizure detection model using single source of features [17].

The life quality of an epilepsy patient will be enormously improved, if a seizure may be predicted before its occurrence. A seizure prediction algorithm evaluates whether a given EEG signal is more similar to the signals before the seizure onset than to those with no following seizure events. Such an accurate prediction model will determine whether an epileptic seizure will happen in the near future. So scientists kept exploring the possibility of accurately predicting the seizure occurrences even a few seconds in advances. Viglione and Walsh carried out the first attempt of predicting epileptic seizures in 1975 [23]. Salant, Y. et al., extracted the spectral features to train an EEG-based epileptic seizure prediction algorithm and successfully predict seizures 1–6 s in advance for 5 patients [24]. A number of recent studies presented much-improved prediction models using the intracranial EEG (iEEG), which requires patients undergoing craniotomies and places electrodes in a selected portion of the brain to measure the patients’ intracranial EEG [25,26,27]. These invasive models may predict epileptic seizures 10–50 min in advances, but are difficult to deploy during patients’ everyday life.

Various other feature extraction technologies have been proposed to describe the EEG signals from different aspects. High Order Spectra (HOS) was the extension of the concept High Order Statistics [28], has been used together with the cumulant features [29] or Continuous Wavelet Transform to detect epileptic seizures. The Discrete Wavelet Transform (DWT) was also applied to extract the frequency domain features of EEG signals for the epileptic seizure detection problem [30]. Intrinsic time-scale decomposition (ITD) is another feature engineering algorithm to represent EEG signals in the time-frequency domain and demonstrated the detection accuracy 95.67% for the normal, inter-ictal and ictal EEG signals [31]. The feature extraction algorithm recurrence quantification analysis (RQA) is a non-linear data analysis method in the chaos theory and has been utilized to train a Support Vector Machine (SVM) model for classifying epileptic seizures in the EEG signals with the accuracy 96.67% [32]. Wang et al. proposed a multi-domain entropy based feature extraction framework and achieved a high classification accuracy 99.25% of the epileptic seizure events [17].

This study integrated 24 different types of features extracted from the scalp EEG signals, and carried out a comprehensive investigation of how these features may be refined to train an accurate epileptic seizure detection model. We further applied the parameters of the optimal model to predict epileptic seizures 5 and 10 s in advances. Our experimental data showed that both the epileptic seizure detection and prediction algorithms achieved at least 99% in accuracies.

## 2. Materials and Methods

### 2.1. Data Description and Sample Extractions

The TUH EEG Seizure Corpus is released by the American Clinical Neurophysiology Society a standard EEG database and this study chose the dataset of 993 scalp EEG recorded files [33,34]. These EEG signals were recorded using 21 electrodes on the scalp of the participants and each electrode generates 250 data points per second (sampling rate 250 Hz) using the averaged reference (AR) channel configuration by a standard ACNS (American Clinical Neurophysiology Society) TCP (Temporal Central Parasagittal) montage [35], as shown in the Table 1.

**Figure 1:**
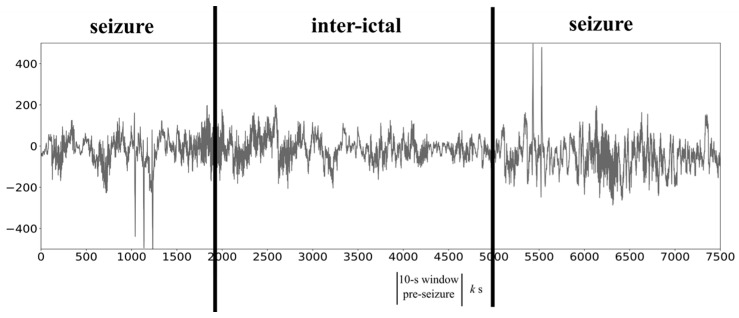
Definitions of seizure samples, inter-ictal samples and pre-seizure samples. This exemplified EEG signal has two seizure onset windows and the inter-ictal window in between.

This dataset annotated 227 seizures for 27 out of 43 patients with diagnosed epilepsy and a data sample was defined as 10-s window of EEG signals extracted from the 993 EEG files, as illustrated in Figure 1. A seizure period is the annotated seizure ictal period in the database TUH EEG, while the inter-ictal period is defined as the region with at least 20 s in length. A seizure sample was a 10-s window randomly extracted from the seizure regions (as illustrated in Figure 1), while an inter-ictal sample was a 10-s window randomly extracted from the inter-ictal period (as illustrated in Figure 1). Both types of samples avoided the 5-s region in the two boundaries. A pre-seizure sample was a 10-s window with a k-second window offset to the seizure period, where the parameter k was set to 0, 5 or 10 s in the study.

### 2.2. Feature Engineering

This study extracted 24 types of features from a given 10-s EEG signal, as described in the Table 2. The number of features extracted from each channel was listed in the column “FpC” of Table 2. These 24 types of features may be grouped as 4 families, i.e., statistical, fractal, entropy and spectral features. Except for the feature type “PSI_RIR”, each of the other feature types has 5 features. Take the feature type “Mean” as an example. An ordered list of voltage values was processed by the Discrete Wavelet Transform (DWT) with the decomposition level 4, and four wavelet coefficients (list) were decomposed from raw EEG. So the feature type “Mean” has the averaged value of the raw EEG data in the time domain and the averaged values of the four wavelet coefficients in time-frequency domain. The spectral feature type “PSI_RIR” has 12 features, as described in the following section. So 127 features were generated from each channel of EEG signals of a sample, and each sample had 127 × 22 = 2794 features in total.

#### 2.2.1. Statistical Features

The 16 statistical feature types were defined as follows. An ordered list of EEG voltage values, defined as *V* = <*V*_1_, *V*_2_, …, *V_n_*>, and the ordered list of the first *k* values were *V*(*k*) = <*V*_1_, *V*_2_, …, *V_k_*>. V was summarized by the averaged (Mean), maximum (Crest), minimum (Trough) and variance (Var) values. The skewness (Skw) of this voltage value list was defined as the asymmetry: $\sum_{i=1}^{n}{\left(V_{i}-\bar{V}\right)}^{3}/\left[(n-1)V_{dev}\right]$, where *V_dev_* was the deviation of this voltage list.

The measurement Kurtosis (Kurt) was defined to measure the “peakedness” of the given list of EEG voltages: $\sum_{i=1}^{n}{\left(V_{i}-\bar{V}\right)}^{4}/\left[(n-1)V_{dev}\right]$ [37]. The absolute peak value of V was defined as Peak. The measurement Root Mean Square (RMS) was the arithmetic mean of the squares of *V_i_*, as in $\sqrt{\sum_{i=1}^{n}V_{i}^{2}}/n$ [38]. And the Peak-to-Average Power Ratio (PAPR) was defined as the Peak divided by RMS of the waveform, and evaluated the difference between the peak and the effective value of the EEG signals [39]. The ratio between RMS and Mean was defined as the Form Factor (FFac), and the total variation (TotVar) was defined as $\sum_{i=1}^{n-1}\left|V_{i+1}-V_{i}\right|/\left[(V_{Max}-V_{Min})(n-1)\right]$.

The Hurst Exponent (also called Rescaled Range statistics) was used to measure the correlations within *V* [40]. The range *R*(*i*) was defined as [max(*V*(*i*)-min(*V*(*i*))] and *S*(*i*) was $\sqrt{\sum_{t=1}^{i}{\left(V_{t}-\bar{V}\right)}^{2}/i}$. The Hurst Exponent (HuExp) was defined as the slope of the line regressed by ln{*R*(*i*)/*S*(*i*)} and ln(*n*).

The statistical self-affinity of V was evaluated by the Detrended Fluctuation Analysis (DFA), and it has been widely used analyze time series data with non-stationary and long-memory property [41]. Hjorth proposed a mathematical method to describe an EEG trace quantitatively [42], which has been widely applied to various EEG-based problems [43,44]. The Hjorth parameter Mobility (HMob) was the ratio of the average frequency and standard deviation, and the parameter Complexity (HComp) described the changes of the signal frequencies.

The Fisher Information (FI) measures the amount of information within a random variable and was widely used to analyze the EEG signals [45].

#### 2.2.2. Fractal Dimension Features

A fractal dimension describes a statistical index of how a complexity pattern changes with the scale, and how similar between the local and global patterns [46]. Fractal dimension performs well on turbulent and irregular time series data and has been widely used to extract quantitative features from biomedical signals, including imaging [47] and EEG [48], etc. Three types of fractal dimension features were used in this study, i.e., Mandelbrot Fractal Dimension (MFD), Petrosian Fractal Dimension (PFD) and Higuchi Fractal Dimension (HFD).

The Mandelbrot Fractal Dimension (MFD) described a fractal dimension in a planar curve as log(*L*)/log(*d*), where L is the sum of the Euclidean distances of all the successive data points in an ordered list, and d is the largest distance between the first point and the others. The Petrosian Fractal Dimension (PFD) simply describes the sign changes in the given ordered list [49]. The Higuchi Fractal Dimension (HFD) measures the signal length and has been applied on various biophysical signals, e.g., ECG [50], MEG [51] and EEG [52], etc. The parameters were set to the same as in [53].

#### 2.2.3. Entropy Features

Entropy is another important aspect for the analysis of biophysical signals [54,55], and four types of entropy features were used in this study.

The classic approximate entropy was refined by the Sample Entropy (SampEn) to measure how complex a time series data is [56]. A smaller value of SampEn suggests that the data is more self-similar. Permutation Entropy (PeEn) was introduced as a simple complexity parameter by the fast calculation formula of neighboring values [57]. PeEn performs similarly to Lyapunov exponents and demonstrates a strong robustness against the various data noises. The Singular Value Decomposition Entropy (SVDEn) evaluates the complexity of the non-stationary bio-signals by calculating the singular value decomposition (SVD) [58]. The irregular signal patterns in the epileptic seizure EEG data may be quantified well by the Spectral Entropy (SEn) [59], so SEn was also employed to describe the EEG signals for the epileptic seizure detection and prediction problems.

#### 2.2.4. Spectral Features

Power Spectral Intensity (PSI) and Relative Intensity Ratio (RIR) were employed to describe the EEG signals in the spectral space. PSI calculates the intensity of each frequency band for a given data list. The frequency range of the EEG signals were usually split into the following frequency bands δ (0.5–4 Hz), θ (4–8 Hz), α (8–13 Hz), β2 (13–20 Hz), β1 (20–30 Hz), γ (30–60 Hz). Let the results of Fast Fourier Transform (FFT) be {*F*_1_, *F*_2_, …, *F_n_*}. Then the list of bands {*f*_1_, *f*_2_, …, *f_m_*} were constructed to describe the frequency boundaries. So the PSI of *k* bands is calculated as $PSI_{k}=\sum_{i=\mathrm{int}(n\times f_{k}/R)}^{\mathrm{int}(n\times f_{k+1}/R)}\left|F_{i}\right|$, and RIR is the density of PSI, defined as $RIR_{k}=PSI_{k}/\sum_{i=1}^{k-1}PSI_{i}$, where *k* ∈ {1, 2, …, *m* − 1} and *R* is the sampling rate [60]. For abbreviation, the two feature types were combined as one feature type PSI_RIR in Table 2.

### 2.3. Experimental Procedure

**Figure 2:**
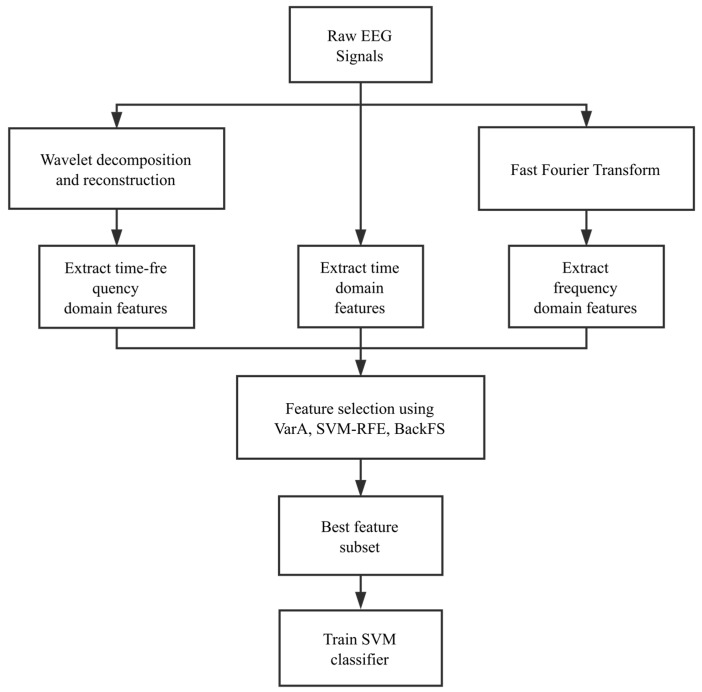
Outline of the experimental procedure. The modules may be roughly grouped as three steps, i.e., feature engineering, feature selection and classification optimization.

This study carried out a series of feature evaluation and model optimization procedures in the following outline, as shown in Figure 2. Compared with intracranial EEG signals, scalp EEG signals are subject to many sources of external disturbances, e.g., the influences of the scalp and skull.

### 2.4. Feature Selection

The step of feature engineering generated 2794 features for each sample, but not all of these features contribute to the discrimination between seizure and inter-ictal samples. These features were evaluated by a series of three consecutive selection algorithms, i.e., VarA, iRFE and BackFS, to ensure the maximal classification accuracy. Firstly, the features with small standard deviations were removed (step VarA). Secondly, the remaining features were ranked by their weights in the trained linear-kernel SVM-RFE model and important features with larger weights were kept for further analysis (Step iRFE). Thirdly, the feature subset was refined by a step BackFS to remove redundancy. The details of each step were described in the following paragraphs.

A feature with a small variation was removed due to its difficulty of being separated at a low data detection resolution, denoted as VarA. That is to say, if a feature remains similar in most of the samples, its discrimination power is questionable. Features were ranked from small to large in their standard deviations, and top 30% of the features were removed from further analysis.

SVM-RFE was demonstrated to perform very well on selecting a subset of features with reasonable performances [61], and was employed on the rest features iteratively, denoted as iRFE. For each iteration, a linear-kernel SVM classification model was trained on the remaining features and the weight of each feature was calculated from the trained SVM model. The feature with the smallest weight was eliminated. Repeat this iteration until the user-specified number of features was met. The parameter feature number was denoted as pNumF, which would be evaluated and optimized in the section Results and Discussion.

Inter-feature redundancy was not investigated in the above two feature selection modules, and an iterative redundancy-removal module BackFS was utilized to further reduce the number of features, as described in the following pseudocode.

Function ***BackFS***(DataMatrix, ClassLabel): Begin Set = null; while FeatureNum(DataMatrix) > 1:   for Feature(*i*) in DataMatrix:     Performance(*i*) = PerformanceMeasurement(DataMatrix\Feature(*i*))   remove the *j*th feature with the largest Performance(j) from DataMatrix   add the largest Performance(j) into Set find the largest one in Set, the subset in that iteration is the best one End.

The variable DataMatrix has the feature data for all the samples, with each row the values of the same feature for all the samples and each column all the feature values for one sample. The variable ClassLabel gives the class assignments of all the samples, and has two different values for a binary classification problem. The function PerformanceMeasurement(DataMatrix) was calculated as the balanced accuracy bAcc = (Sn + Sp)/2 using the 10-fold cross validation strategy of the binary classifier linear-kernel SVM. The denotion DataMatrix\Feature(i) was the data matrix after removing the row of the feature Feature(i).

### 2.5. Classification Performance Measurement

This study used the following widely-used performance measurements for a binary classification algorithm, i.e., sensitivity (Sn), specificity (Sp) and accuracy (Acc) [61]. For the seizure detection problem, seizure samples are defined as the positive samples, while the inter-ictal samples are negative ones. For the seizure prediction problem between pre-seizure and inter-ictal samples, pre-seizure samples are positives while inter-ictal samples are negatives. Sn is defined as the percentage of correctly predicted positive samples, while Sp is the percentage of correctly predicted negative samples. Acc is the percentage of overall correctly predicted samples. Another measurement bAcc was introduced as (Sn + Sp)/2 for the dataset with in-balanced classes [62]. All these performance measurements were calculated by the 10-fold cross validation strategy.

The default classifier linear-kernel SVM was compared with 5 other classification algorithms. Logistic Regression (LR) is a regression-based binary classifier [63] while Naïve Bayes (NBayes) is a parameter-independent Bayesian classifier [64]. Both LR and NBayes estimate the probabilities of whether a sample belongs to a class. Random Forest (RF) is a randomized forest based classifier [65]. Gradient Boosting (GBoost) classifier is a greedy searching strategy to find the best solution [66]. K Nearest Neighbor (KNN) is a simple parameter-independent classifier [67].

## 3. Results

This study extracted a comprehensive list of 24 feature types from the EEG signals and investigated the EEG-based seizure detection problem. These feature types may be roughly grouped as four feature families, i.e., statistical, fractal, entropy and spectral features. So it would be interesting to evaluate how each feature type and their combinations contribute to the seizure detection performances. Then we will select the best set of features to build the seizure detection model. Finally we will tackle the challenge of predicting the seizure onset before it really happens.

### 3.1. How the 24 Feature Types Contribute Individually

**Figure 3:**
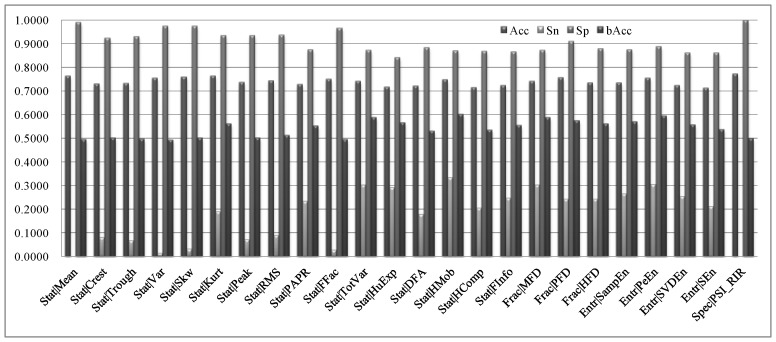
Binary classification performances of each of the 24 feature types. Names of the feature types were defined in the Table 2, and the prefix “Stat|”, “Frac|”, “Entr|” and “Spec” represent the feature families Statistical, Fractal, Entropy and Spectral, respectively.

This study firstly evaluated how each of the 24 feature types contributes to the classification performances, as shown in Figure 3. The maximal bAcc = 60.15% was achieved by the statistical feature type HMob, which is the Hjorth mobility parameter, while the minimal bAcc = 49.35% was achieved by the statistical feature type Var. The fractal features achieved the best averaged bAcc = 57.47%, which is slightly better (0.99%) than that of the entropy features. The average bAcc of all the 24 feature types is only 54.07%, which slightly better than a random guess. Figure 3 also suggested that the detection model using single feature type performed much worse on the seizure samples than the inter-ictal ones. So we hypothesized that the integration of multiple feature types may perform better.

### 3.2. Pairwise Orchestration of the 24 Feature Types

**Figure 4:**
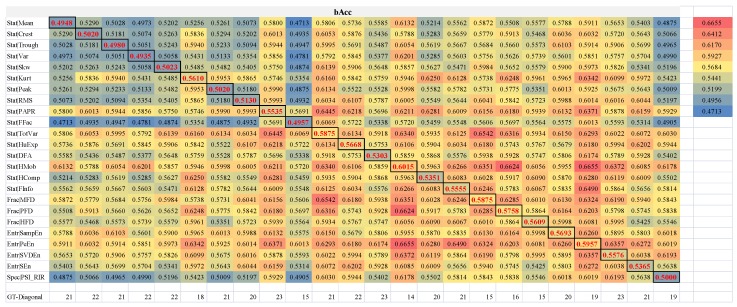
Performance measurement bAcc of the pairwise feature types. The heatmap was colored from blue (minimal bAcc = 0.4713) to red (maximal bAcc = 0.6655). The diagonal grids with red font and black box are pairs of the same feature types, e.g., the top left box gives the bAcc = 0.4948 of a pair of feature types (Stat|Mean, Stat|Mean). The columns and rows are in the same orders of all the 24 feature types. The row “GT-Diagnal” gives the numbers of pairwise orchestrations of feature types that achieved better bAcc than the diagonal grids.

All the models using a single feature type were outperformed by an orchestration with one other feature type, as shown in Figure 4. The diagonal grids illustrated the performance measurements bAcc of a pair of the same feature types, i.e., only one feature type was utilized. While the other grids illustrated how two different feature types collaborated with each other. The bottom row “GT-Diagonal”. For any of the 24 feature types, its orchestrations with at least half of the other feature types outperformed this single feature type. All the other 23 feature types orchestrated with the feature type Stat|PAPR outperformed the model using this feature type only. Another two feature types Stat|DFA and Entr|SVDEn demonstrated the same observations. The maximal improvement 25.65% in bAcc over a single feature type was achieved by an orchestration of Stat|Var with Stat|HMob compared with the single feature type Stat|HMob.

### 3.3. How the Best Model Was Achieved

**Figure 5:**
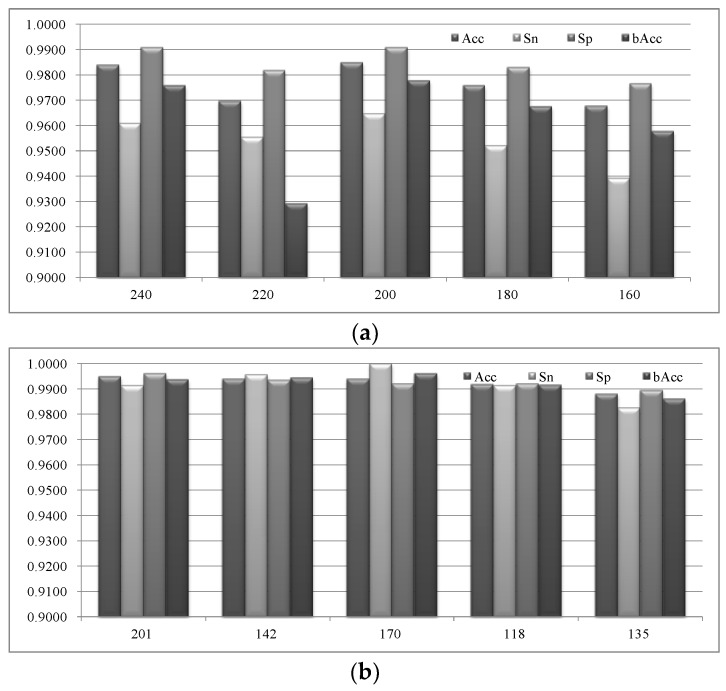
The classification performances of the linear-kernel SVM classifier with different feature numbers. (**a**) The horizontal axis is the number of features (parameter *pNumF*), while the vertical axis is the classification performance value of the four measurements Acc/Sn/Sp/bAcc. (**b**) The feature subset was further filtered by the module BackFS to remove inter-feature redundancies.

The above experimental data showed the necessity of integrating multiple feature types to improve the seizure detection model. So we carried out our three-step feature selection procedure VarA/iRFE/BackFS on all the 24 feature types to get an optimal feature subset for the seizure detection model, as shown in Figure 5. After removing the features with little variations (step VarA), we evaluated whether the two steps iRFE and BackFS are necessary. The classification performances were calculated using the linear-kernel SVM classifier by the 10-fold cross validation strategy.

The best value for the parameter pNumF is 200. Firstly, the feature selection module iRFE selected the best feature subset when the parameter pNumF is 200, as shown in Figure 5a. Both of the two measurements Acc and bAcc reached their best values at pNumF = 200. But the sensitivity (Sn) was still not very accurate and didn’t reach 97.00% for all the choices of pNumF, i.e., 160, 180, 200, 220, and 240. Then we applied the redundancy removal step BackFS on the feauture subset, and significantly improved the seizure detection model, as shown in Figure 5b. The value 200 is still the best choice for the parameter pNumF. We may see that the three measurements Sn/Sp/bAcc reached the best values for pNumF = 200, but Acc was slightly smaller (0.10%) than that of pNumF = 240. Considering that the model of pNumF = 200 achieved Sn = 100.00% and Sp = 99.22%, while the model of pNumF = 240 achieved Sn = 99.13% and Sp = 99.61%, it may be preferred to have a minor increased false alarm rate with an accurate seizure capture capability.

The best model used 170 features after the redundancy was removed from the 200 features generated from iRFE, as shown in Figure 5b. Montage 8 contributed 13 features to the final feature subset, while both of the montages 17 and 18 contributed 11 features. It’s interesting to see that the montage 8 on the left side of the brain contributed the largest number of features to the best model. But the next two best montages 17 and 18 were located in the left and right sides, respectively. So we summed the feature contributions of the two brain sides, and observed little difference (two-tailed unpaired *t*-test *p* value = 0.6324) between the contributed feature numbers of the montages from the left and right sides of the brain. Actually, the left and right sides of the brain contributed 88 and 82 features to this best model.

So the following sections will use 200 as the default value for the parameter pNumF.

### 3.4. The Performance of Models Using Different Classifier

**Figure 6:**
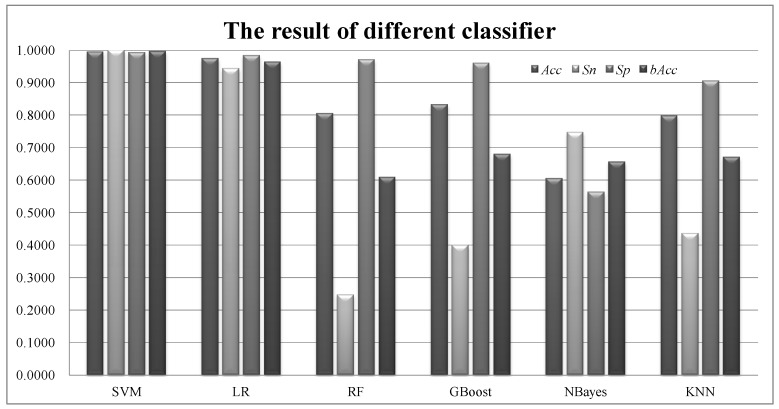
Binary classification performances of different classifiers on detection epileptic seizures using the 22-channel EEG signals. All the classifiers were provided in Python with the default parameters.

We further evaluated whether other classifiers may achieve better classification performances on the best feature subset, as shown in Figure 6. The classifier SVM was used in this study and demonstrated a significant improvement over the four classifiers, i.e., RF, GBoost, NBayes and KNN. The best of these four classifiers didn’t achieve bAcc = 67.98% better than 70%. The classifier LR performed very well in bAcc = 96.33%, but the best classifier SVM outperformed LR by an improvement of 3.28% in bAcc. SVM achieved Sn = 100% in detecting the positive samples while Sp = 99.22% for detecting the negative controls.

The deep supervised learning algorithm convolutional neural network (CNN) have already been evaluated for the classification performances on the EEG signal based epileptic seizure detection problem. CNN was compared with the existing classification algorithms, including Linear Discriminant Classifier (LDC) and Logistic Classifier (LOGLC), etc. The model was optimized to achieve 91.92% classification accuracy [68]. Thodoroff et al. conducted a comprehensive evaluation of how to optimize the deep convolutionary neural network (ODCN) but didn’t outperform the algorithm proposed in our study [69]. In the last year, another CNN classifier was applied to detect epileptic seizures and achieved the accuracy 88.7% [70]. So the deep learning model CNN doesn’t achieve better results than the proposed method.

So our feature selection procedure works best with the SVM classifier on analyzing the EEG signal data.

### 3.5. Predicting an Epilepsy Seizure before It Happens

**Figure 7:**
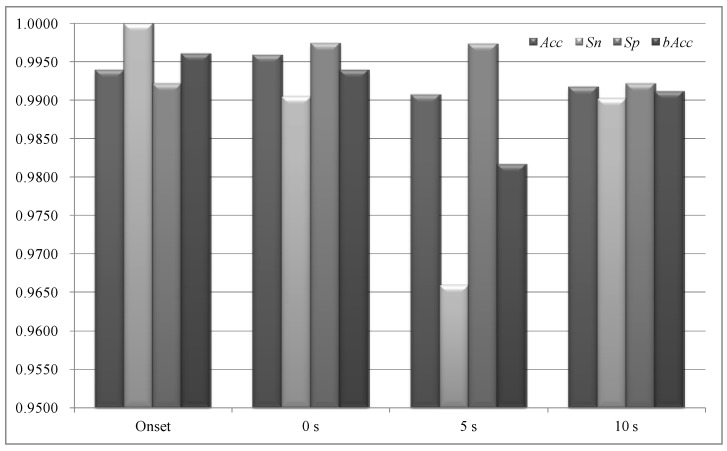
Predicting epileptic seizures before their onsets. The binary classification performances were evaluated using Acc, Sn, Sp and bAcc.

The same modeling procedure was applied to the problem of predicting epileptic seizures before their onsets, as shown in Figure 7. The two datasets “Onset” and “0 s” are different in that the positive samples of the dataset “Onset” were extracted from the seizure occurring time, while the positive samples of “0 s” were extracted right before the seizure onset. The positive samples of the datasets “5 s” and “10 s” were extracted 5 and 10 s before the annotated onset time of the epileptic seizures. Our data showed that there was a slight decrease in bAcc for predicting epileptic seizures in advances, but the overall performances were acceptable, with bAcc > 98% for all the three cases of seizure predictions. Due to the fact that the dataset didn’t provide EEG signals more than 10 s for most patients, this study didn’t evaluate our experimental procedure on predicting epileptic seizures with longer time before onset. A recent study presented a proof-of-concept experiment of predicting epileptic seizures 15 min before happening using the invasive intracranial EEG signals and achieved 69% in sensitivity, which left large room for improvements [71].

## 4. Discussion

A comparison was carried out with EEG-based epileptic seizure detection studies, mostly using the invasive EEG datasets. This study used the latest non-invasive scalp EEG signals which was published in 2017 [33,34], and very few studies have been conducted on this dataset.

The EEG signal differences between epileptic seizures and normal controls were significant enough for building an EEG-based seizure detection or prediction problems. Salant et al. explored the possibility of predicting seizures 1–6 s in advance using the two-channel EEG data and didn’t calculate the prediction accuracy [24]. Kiymik et al. compared how to evaluate the difference between the EEG signals of a normal child and a pediatric epileptic seizure patient using the features of wavelet transform (WT) and short-time fourier transform (STFT) [72]. Li et al. investigated the inter-seizure non-linear similarity of the EEG signals in the phase space [73].

Most of the existing studies didn’t outperform this study in the epileptic seizure detection problem. Guler et al. extracted Lyapunov exponents from the single-channel intracranial EEG signals and trained a recurrent neural network model with 96.79% in accuracy [74]. Polat et al. utilized the fast fourier transform features to train a decision tree classifier and achieved the 10-fold cross validation accuracy 98.72% for detecting hippocampus probed EEG signals from the scalp EEGs of healthy controls [19]. Ocak, Hasan extracted the approximate entropy and discrete wavelet transform features from the EEG signals, and achieved the best accuracy 96% [30].

Three studies achieved slightly better accuracies than this study’s accuracy 99.4%. Polat et al. utilized the Principal Component Analysis (PCA) to the fast fourier transform features and achieved almost 100% in accuracy [75]. Another study employed the power spectral features of EEG signals and achieved 99.6% in accuracy using the Fisher discriminant linear algorithm [76]. Hilbert–Huang Transform (HHT) was proposed to describe the EEG signals using the intrinsic mode functions (IMFs) and improved the SVM classification model to the accuracy 99.85% [77]. But all these three studies were based on the invasive seizure EEG signals, which have much better signal quality than this study. And their prediction accuracies need to be verified on the non-invasive seizure signals before their applications in the everyday life of epileptic patients.

Various other feature types were extracted from the EEG signals for the epileptic seizure detection problem. A fully discrete wavelet transform, the tunable-Q factor wavelet transform (TQWT), was applied to the EEG signals but the detection models were trained separately for each of the six patients [78]. Li et al. trained the neural network ensemble classifier on the discrete wavelet transform (DWT) with 98.78% in accuracy [79]. Wang et al. integrated multiple domains of feature extraction types and achieved a stable 99.25% in accuracy by the 10-fold cross validation [17]. The seizure onset EEG signals of these studies were recorded invasively from the epileptic patients.

A recent study extracted the wavelet-based features from the EEG signals and trained an SVM model with the radial basis function (RBF) kernel with 10-fold cross validation accuracy 96.87% [80]. Our work further improved this model to the accuracy 99.4% using the same validation strategy.

Most of the existing studies didn’t carry out a feature selection step and this study suggested that the features extracted from the EEG signals may need to be further refined before fed into the classification models. Figure 5 illustrated that at least 15% of the EEG-extracted features may be removed to further improve the epileptic seizure detection accuracy. The best model selected 170 from the 200 EEG-extracted features and achieved 99.40% in detection accuracy.

## 5. Conclusions

This study integrated 24 feature types extracted from the scalp EEG signals, and accurately detected epileptic seizures after reducing 2794 features to 170. The same feature selection and classification procedure also performed very well on predicting epileptic seizures a few seconds before their onsets. Such models may facilitate the epileptic seizure monitoring and early warning and significantly improve the patients’ life qualities. The new technique Eddy current pulsed thermography (ECPT) will also be tested in the EEG analysis for its capability of automatically splitting the EEG signals into useful data sources [81]. But due to the fact that the EEG signals may be recorded both intracranially and on the scalp, a detailed investigation may be necessary to optimize how the technique will be tuned [81,82].

The main advantages of our work are that we used the non-invasively collected scalp EEG data and optimized the model on the population level. Most of the existing studies utilized the intracranial EEG (iEEG) which may not fit the everyday living environment [25,71]. A model analyzing the non-invasive scalp EEG signals may help the patients easily manage their everyday lives. Our work also presented a proof-of-concept experiment of patient-independent seizure prediction. Many of the existing studies investigated the patient-specific seizure prediction problem and trained a model for each of the patients in their studies [83,84,85]. Such a modeling strategy has a merit of establishing patient-specific patterns, but has to wait for the data of multiple seizures before a model can be provided for a patient.

The major disadvantage of our work is the intensive computational requirement. We are planning to solve this challenge by both coding optimization and GPU-based parallelization, so that our model may generate the results within an acceptable time frame. Due to the limitations in the public datasets, this study only investigated the seizure prediction problem 10 s before the seizure events. We plan to work with our clinical collaborators to collect 24-h continuously-monitoring scalp EEG data, so that we may explore the possibility of predicting a seizure much earlier than 10 s before its happening.

We will also collaborate with the clinicians to test predicting seizures before their happening in the real world. Our work may significantly soothe the anxiety of a patient by warning the coming of a seizure at least a few seconds earlier.

## Figures and Tables

**Table 1:** Standard definitions of the ACNS TCP montages. Columns “Ref1” and “Ref2” give the channel IDs, and the probing locations of these channels may be found in the [36].

Montage	Name	Ref1	Ref2
0	FP1-F7	FP1	F7
1	F7-T3	F7	T3
2	T3-T5	T3	T5
3	T5-O1	T5	O1
4	FP2-F8	FP2	F8
5	F8-T4	F8	T4
6	T4-T6	T4	T6
7	T6-O2	T6	O2
8	A1-T3	A1	T3
9	T3-C3	T3	C3
10	C3-CZ	C3	CZ
11	CZ-C4	CZ	C4
12	C4-T4	C4	T4
13	T4-A2	T4	A2
14	FP1-F3	FP1	F3
15	F3-C3	F3	C3
16	C3-P3	C3	P3
17	P3-O1	P3	O1
18	FP2-F4	FP2	F4
19	F4-C4	F4	C4
20	C4-P4	C4	P4
21	P4-O2	P4	O2

**Table 2:** Summary of the 24 feature types extracted from each of the 22 channels of a 10-s sample. Column FpC gives the number of features extracted from the 10-s window per channel.

Family	Type	Description	FpC
Statistical	Mean	Average	5
	Crest	Maximum value	5
	Trough	Minimum value	5
	Var	Variance	5
	Skw	Skewness	5
	Kurt	Kurtosis	5
	Peak	Peak value	5
	RMS	Root Mean Square	5
	PAPR	Peak-to-Average Power Ratio	5
	FFac	Form Factor	5
	TotVar	Total Variation	5
	HuExp	Hurst Exponent	5
	DFA	Detrended Fluctuation Analysis	5
	HMob	Hjorth Parameters: Mobility	5
	HComp	Hjorth Parameters: Complexity	5
	FInfo	Fisher information	5
Fractal	MFD	Mandelbrot Fractal Dimension	5
	PFD	Petrosian Fractal Dimension	5
	HFD	Higuchi Fractal Dimension	5
Entropy	SampEn	Sample Entropy	5
	PeEn	Permutation Entropy	5
	SVDEn	SVD Entropy	5
	SEn	Spectral Entropy	5
Spectral	PSI_RIR	Power Spectral Intensity, and the relative intensity Ratio	12
